# The clinical characteristics of dementia with Lewy bodies and a consideration of prodromal diagnosis

**DOI:** 10.1186/alzrt274

**Published:** 2014-07-21

**Authors:** Paul C Donaghy, Ian G McKeith

**Affiliations:** 1 Level 3, Biomedical Research Building, Institute for Ageing and Health, Campus for Ageing and Vitality, Newcastle University, Newcastle NE4 5PL, UK

## Abstract

Dementia with Lewy bodies (DLB) is the second most common type of degenerative dementia following Alzheimer’s disease (AD). DLB is clinically and pathologically related to Parkinson's disease (PD) and PD dementia, and the three disorders can be viewed as existing on a spectrum of Lewy body disease. In recent years there has been a concerted effort to establish the phenotypes of AD and PD in the prodromal phase (before the respective syndromes of cognitive and motor impairment are expressed). Evidence for the prodromal presentation of DLB is also emerging. This paper briefly reviews what is known about the clinical presentation of prodromal DLB before discussing the pathology of Lewy body disease and how this relates to potential biomarkers of prodromal DLB. The presenting features of DLB can be broadly placed in three categories: cognitive impairment (particularly nonamnestic cognitive impairments), behavioural/psychiatric phenomena (for example, hallucinations, rapid eye movement sleep behaviour disorder (RBD)) and physical symptoms (for example, parkinsonism, decreased sense of smell, autonomic dysfunction). Some noncognitive symptoms such as constipation, RBD, hyposmia and postural dizziness can predate the onset of memory impairment by several years in DLB. Pathological studies of Lewy body disease have found that the earliest sites of involvement are the olfactory bulb, the dorsal motor nucleus of the vagal nerve, the peripheral autonomic nervous system, including the enteric nervous system, and the brainstem. Some of the most promising early markers for DLB include the presence of RBD, autonomic dysfunction or hyposmia, ^123^I-metaiodobenzylguanidine cardiac scintigraphy, measures of substantia nigra pathology and skin biopsy for α-synuclein in peripheral autonomic nerves. In the absence of disease-modifying therapies, the diagnosis of prodromal DLB is of limited use in the clinic. That said, knowledge of the prodromal development of DLB could help clinicians identify cases of DLB where the diagnosis is uncertain. Prodromal diagnosis is of great importance in research, where identifying Lewy body disease at an earlier stage may allow researchers to investigate the initial phases of dementia pathophysiology, develop treatments designed to interrupt the development of the dementia syndrome and accurately identify the patients most likely to benefit from these treatments.

## Introduction

**Figure 1:**
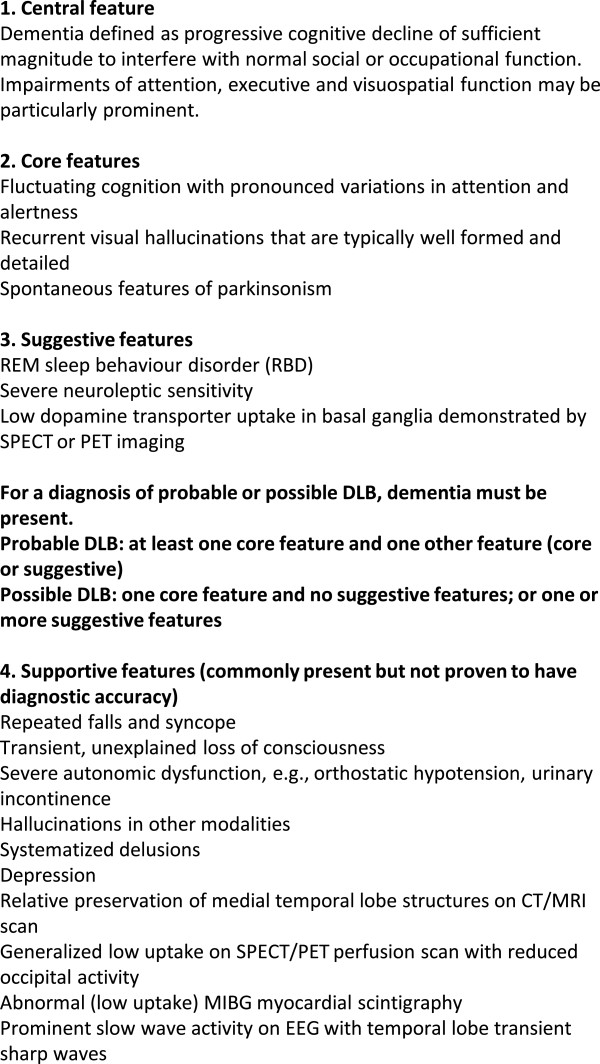
**Diagnostic criteria for dementia with Lewy bodies.** CT, computed tomography; DLB, dementia with Lewy bodies; EEG, electroencephalogram; MRI, magnetic resonance imaging; MIBG, metaiodobenzylguanidine; PET, positron emission tomography; REM, rapid eye movement; SPECT, single photon emission computed tomography. Adapted from [2].

Dementia with Lewy bodies (DLB) is the second most common type of degenerative dementia following Alzheimer’s disease (AD). DLB accounts for around 4.2% of all dementia diagnosed in the community, and 7.5% of those under secondary care [1]. The characteristic features of DLB are spontaneous parkinsonism, recurrent visual hallucinations, fluctuating cognition, rapid eye movement sleep behaviour disorder (RBD), severe sensitivity to antipsychotic medications and reduction in striatal dopamine transporters on single photon emission computed tomography (SPECT) or positron emission tomography (PET) (Figure 1) [2]. The pattern of neuropsychological deficits seen in DLB is different to those in AD, with less marked memory impairment and more severe impairments of visuospatial, attentional and frontal-executive function [3].

**Figure 2:**
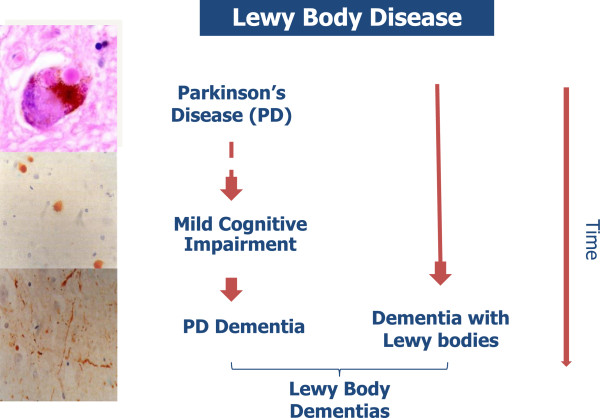
**Nomenclature of Lewy body diseases.** Parkinson’s disease dementia is diagnosed when cognitive impairment develops a year or more after the onset of parkinsonism. Dementia with Lewy bodies is diagnosed when cognitive symptoms appear without parkinsonism or less than 1 year after the onset of parkinsonism.

The clinical features of DLB and Parkinson’s disease dementia (PDD) are similar [2,4]. Based on international consensus, DLB is diagnosed when cognitive impairment precedes parkinsonism or begins within a year of parkinsonism. PDD is diagnosed when parkinsonism precedes cognitive impairment by more than 1 year (Figure 2) [2]. DLB and PDD are now recognised in the *Diagnostic and Statistical Manual of Mental Disorders*, where they are respectively coded as ‘Major and Mild Neurocognitive Disorder with Lewy Bodies’ and as ‘Major and Mild Neurocognitive Disorder due to Parkinson’s Disease’ [5].

As with Parkinson’s disease (PD) and PDD, the main pathological lesions seen in DLB are Lewy bodies (LBs) and Lewy neurites, both containing α-synuclein (αSyn) [6]. The pattern of distribution of LB pathology in DLB, PD and PDD as seen at end stage is very similar, although cortical involvement may occur earlier in DLB and brainstem involvement may be minimal [7-9]. The three disorders can be viewed as existing on a spectrum of LB disease [10], suggesting that studies of symptom development and pathology in PD, although not identical, are probably highly relevant to any consideration of the early stages of DLB.

In recent years there has been a concerted effort to establish the phenotypes of AD and PD in the prodromal phase, which is defined as the period between the onset of the earliest symptoms and the development of the full clinical syndrome. Evidence of the prodromal presentation of DLB is also emerging [11]. Criteria for the clinical diagnosis of DLB have high specificity but low sensitivity [12]. In the move towards earlier diagnosis, biomarkers of LB disease may be necessary to optimise diagnostic accuracy. This paper briefly considers what is known about the clinical presentation of prodromal DLB before discussing the pathology of LB disease and how this relates to potential biomarkers of prodromal DLB. The paper will then discuss how this knowledge can be applied to current clinical and research practice.

## Symptoms of prodromal dementia with Lewy bodies

**Figure 3:**
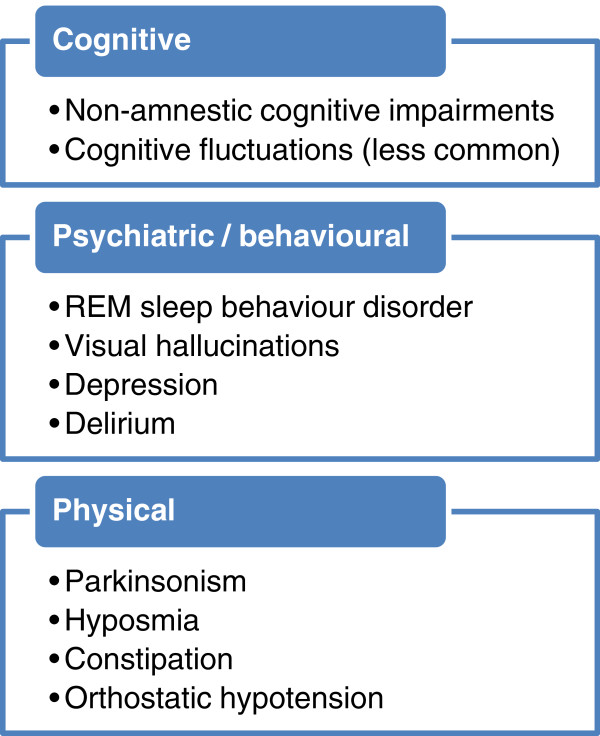
**Examples of presenting symptoms of dementia with Lewy bodies.** REM, rapid eye movement.

The presenting features of DLB can be broadly placed in three categories (Figure 3): cognitive impairment; behavioural/psychiatric phenomena; and physical symptoms.

### Cognitive impairment

Mild cognitive impairment (MCI) is conceived as a state intermediate between normal cognitive function and dementia [13]. People with MCI are at increased risk of developing dementia [14], leading to MCI being considered a prodromal phase of dementia [15]. A proportion of MCI cases that convert to dementia may develop DLB, although rates have varied from around 5% to around 25% across different studies [16-19]. Some of this variation is likely to be due to recruitment criteria for the studies; for example, amnestic MCI largely from primary care, 5.6% DLB [17]; MCI from a tertiary referral centre, 28.4% DLB [18]. DLB can be preceded by amnestic or nonamnestic cognitive impairment, although cases involving nonmemory domains (that is, attention/executive, visuospatial or language) are more likely to progress to DLB than single-domain amnestic MCI [19,20].

Fluctuating attention and cognition is a core feature of DLB [2], but evidence so far suggests that fluctuations are the least common core symptom in the prodromal phase (present in 2/7 cases [21] and 3/9 cases [22] in two longitudinal studies). Delirium and transient disturbances of consciousness, however, are reported as prodromal features of DLB and may represent the earliest manifestations of cognitive fluctuation [23].

### Behavioural/psychiatric phenomena

Importantly, DLB may initially present with noncognitive symptoms such as visual hallucinations, depression and RBD [24]. RBD is a parasomnia characterised by the enactment of dreams (for example, punching, kicking, shouting out) that often results in injury. Longitudinal studies of RBD have shown that up to 93% of cases go on to develop a synucleinopathy – PD, PDD, DLB or multiple system atrophy, a clinically and pathologically distinct non-LB synucleinopathy [25] – if followed up for a sufficient number of years [26-28].

Retrospective studies have found that visual hallucinations and RBD can be present in around one-half of DLB cases prior to or around the onset of memory loss [24,29,30]. Anxiety and depression were present in around one-quarter of patients [24,29]. Retrospective case–control studies have found that a history of depression [31] or delirium [23] prior to the diagnosis of dementia is more common in DLB than AD, although both delirium and depression are probably too common in the normal older person to be useful biomarkers in isolation.

### Physical symptoms

Parkinsonism is a core feature of DLB, and may be a presenting symptom in around one-quarter of patients [24]. αSyn deposition in the olfactory bulb, brainstem and peripheral nervous system in LB disorders is associated with a variety of physical symptoms, such as decreased sense of smell (hyposmia), constipation, orthostatic dizziness and increased salivation [24].

Some noncognitive symptoms of DLB such as constipation, hyposmia and postural dizziness can predate the onset of memory impairment by years in DLB [29]. In the earliest clinical phase of DLB, patients may therefore be expected to present with one or, more probably, a combination of these symptoms in mild form. Based on the presence of these symptoms one could suppose that a person has prodromal LB disease, even in the absence of cognitive dysfunction, but given that these are common complaints in older people, they are in themselves likely to be very nonspecific predictors. Given this nonspecificity, a combination of several symptoms along with other biomarkers may be necessary to identify those with prodromal DLB.

## Neuropathology of dementia with Lewy bodies

**Figure 4:**
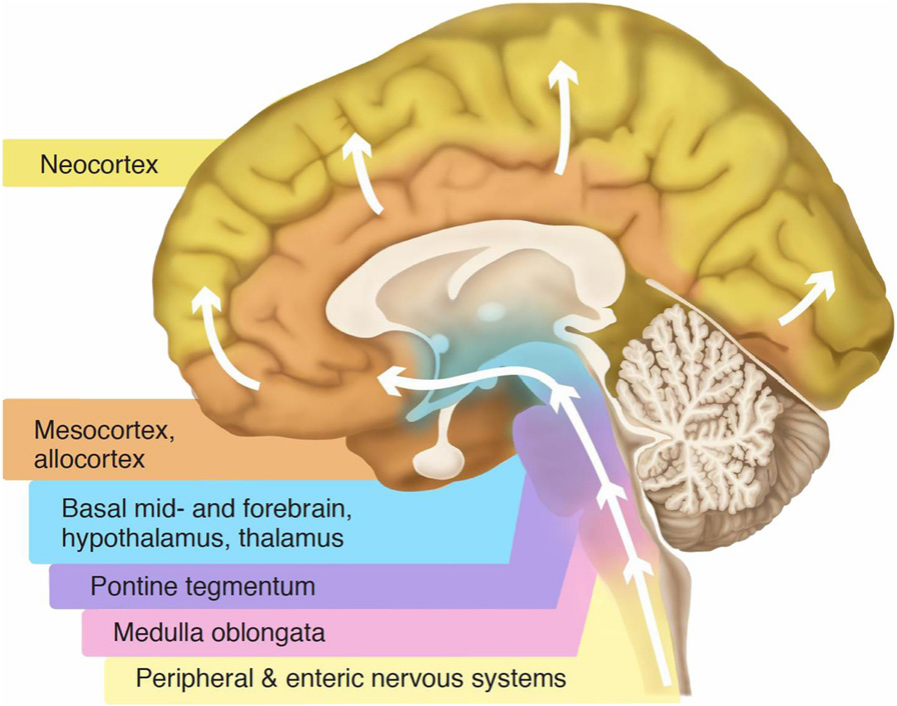
**Progression of Lewy body pathology.** Schematic diagram of the pattern of alpha-synuclein deposition as hypothesised by Braak and colleagues [32]. Deposition is first seen in the peripheral autonomic nervous system, including the enteric nervous system. This is then followed by deposition in the brainstem, ascending to the limbic system and, finally, the neocortex. Illustration from [88], available under creative commons license.

Prior to any discussion of the probable biomarkers of prodromal DLB, the temporal development of LB pathology must be considered. Much of the work on the pathology of LB disease comes from research in PD. Just over a decade ago, Braak and colleagues proposed a staging system for Lewy pathology in PD [32]. In a sample of brains from PD patients and asymptomatic individuals with LB disease, they found that all could be classified into one of six stages of disease development. At the earliest stage (stage 1), LB pathology was confined to the dorsal motor nucleus of cranial nerves IX/X and the intermediate reticular zone of the medulla. Over the subsequent stages, LB pathology ascended sequentially through the pons, midbrain and subcortical structures to finally affect the neocortex itself in stages 5 and 6 (Figure 4). A key feature of Braak staging was that the sequential ascent of LB pathology from the brainstem to the neocortex was common to all cases – that is, the cortex was not involved in every case; but where the cortex was affected, so were lower structures such as the limbic system and brainstem. Others have observed that LB pathology does not necessarily follow this pattern of distribution, particularly when cases are sampled from the general population, in which LB pathology can be found in higher centres such as the neocortex, despite sparing of lower regions [7,33-35]. Nevertheless, it is clear that some structures are particularly susceptible to LB pathology and may potentially act as sentinels for the development of LB disease: the olfactory bulb [7], the dorsal motor nucleus of the vagal nerve and other brainstem structures [32] and the peripheral autonomic nervous system [36,37], including the enteric nervous system [38].

The pattern of deposition of Lewy pathology in DLB cases seen at autopsy is similar to that of PD/PDD [7,8]. Dementia is the clinical expression of widespread and relatively advanced brain disease. Unsurprisingly, then, the majority of DLB cases are found to have an advanced Braak stage, with cortical involvement [7,8]. The actual amount of cortical LB pathology does not necessarily correlate with clinical dementia severity [39], and significant cortical pathology can occur in the absence of clinical symptoms [33,40]. One explanation for this is that LBs do not directly cause cell dysfunction and death. Presynaptic accumulation of αSyn and resultant synaptic dysfunction has been put forward as a causative factor of neurodegeneration in LB disease but, like LB deposition, this cannot yet be measured *in vivo*[41].

Postmortem examination of brains from people who suffered from DLB is informative to a degree about the pathology of the later stages of disease, but less so about the earlier stages of disease development. Findings from studies of early PD and LB disease without any clinical symptoms (incidental LB disease) may be used to hypothesise about the early pathological development of DLB.

Incidental LB disease with cortical involvement has been suggested to be more likely to progress to DLB than PD [33]. If so, the profile of structural and functional brain changes that can be detected *in vivo* during the prodromal phases of LB disease will probably also vary between DLB and PD, reflecting differences in the underlying pathology. Some pathological differences between DLB and PDD have been identified [42]. DLB is associated with higher amyloid plaque deposition in the striatum [43,44], more frequent αSyn deposition in the CA2/3 area of the hippocampus [45] and significantly higher 5-HT_1A_ receptor density in the frontal cortex [46]. Compared with PD, DLB demonstrates less marked cell loss in the substantia nigra, and a relative lack of D_2_ receptor upregulation in the striatum [47].

Although LB pathology is the pathological hallmark of DLB, other types of pathology may interact with LB pathology, or may potentially mimic the DLB phenotype in the absence of significant LB pathology. In community-based cohorts, dementia is most often associated with mixed pathology including AD, vascular and LB pathology [48]. MCI in PD is associated with heterogeneous pathology [42]. Coexisting LB and AD pathology (amyloid-beta (Aβ) and tau) is frequently found in DLB at postmortem [49,50]. The importance of AD pathology in DLB is not yet clear. In amyloid PET imaging studies, LB disease groups have lower mean amyloid ligand binding than AD groups. Many cases have normal levels of amyloid binding, although amyloid deposition is more common in DLB than PDD and is relatively rare in PD, suggesting that Aβ may be associated with an increased risk of dementia in LB disease [51]. The presence of AD pathology may reduce the likelihood of expression of the typical DLB phenotype [52,53]. In some cases of LB disease, LBs are predominantly confined to the amygdala [54]. This pattern of deposition is common in AD, and may represent a different clinicopathologic process to PD/PDD/DLB [7,55].

## A hypothetical biomarker profile of prodromal dementia with Lewy bodies

**Figure 5:**
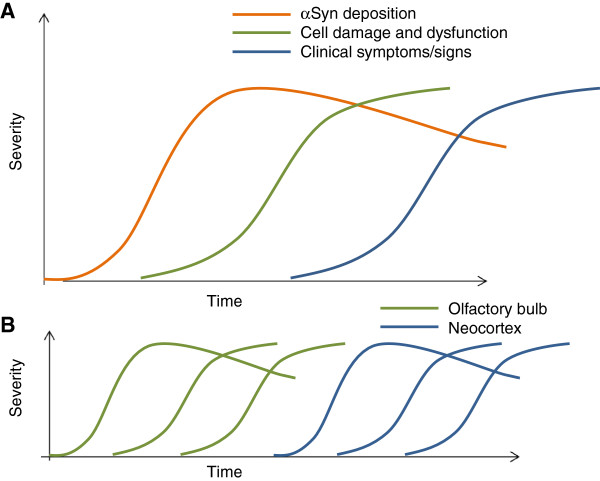
**Hypothetical timelines of biomarker development. (A)** Biomarker development in dementia with Lewy bodies (DLB). This hypothesis mirrors that proposed by Jack and colleagues in Alzheimer’s disease [56]. The first biomarkers of DLB will be markers of alpha-synuclein (αSyn) deposition (for example, from skin biopsy). αSyn deposition probably decreases later in the disease process following cell death [40]. This would then be followed by markers of cell damage or death (for example, loss of dopamine transporters in the striatum on single photon emission computed tomography or positron emission tomography) and then clinical symptoms/signs (for example, parkinsonism). **(B)** Biomarkers in two different sites. In this hypothetical representation, αSyn deposition, cell damage and loss and the development of symptoms (hyposmia) all occur in the olfactory bulb prior to the development of αSyn deposition in the neocortex.

Jack and colleagues put forward a model of dynamic biomarkers for AD, with markers of Aβ deposition sequentially followed by markers of tau-mediated neuronal injury and dysfunction, changes in brain structure, abnormalities in tests of memory and, finally, a decline in day-to-day function [56]. This hypothesis has been criticised due to emerging evidence against the amyloid cascade hypothesis [57]. Nevertheless, the idea of a process beginning with abnormal protein deposition followed by cell damage or death and then loss of function is a useful conceptual base from which to consider the likely development of biomarkers in DLB (Figure 5A). DLB is associated with a relative lack of structural brain changes compared with AD [58] and structural change can be presumed to be less likely and therefore harder to detect in the earliest stages of disease. The additional effects of Alzheimer pathology may well operate to a greater or lesser extent in many subjects but these have not been incorporated into the schematic.The process of abnormal protein deposition–cell damage–functional decline will occur at different times in different areas (Figure 5B), with both the peripheral and central nervous systems involved early in DLB. For example, measureable loss of function may occur in the olfactory system before significant protein deposition has occurred in higher cortical areas. The following paragraphs examine possible biomarkers of protein deposition, cell damage and functional decline to assess their potential usefulness in prodromal DLB in relation to what is known about the pathology of LB disease.

### Biomarkers of protein deposition

*In vivo* measurement of Aβ, the putative pathological hallmark of AD, is possible with cerebrospinal fluid Aβ levels and amyloid PET imaging. No radioligands are yet available to image αSyn *in vivo*, although some are in development [59]. Several studies have found reduced cerebrospinal fluid αSyn in established DLB, although other studies have found no such differences [60]. This heterogeneity of results may reflect methodological differences in the measurement of αSyn, or cerebrospinal fluid contamination with red blood cells, which are relatively rich in αSyn [60]. The utility of blood and cerebrospinal fluid biomarkers in the diagnosis of DLB remains unresolved. There has been no research into the use of such markers in the prodromal stage of DLB.

Some of the earliest sites of LB deposition include the olfactory bulb [7,32] and the enteric nervous system [38,61]. Biopsies from these sites are a possible route to detect αSyn deposition *in vivo*[62,63] with potential for early diagnosis, although the invasiveness of these techniques could limit their application [64].

More recently, Wang and colleagues used a new technique to measure the proportion of peripheral autonomic nerve fibres containing αSyn in skin biopsies [65]. In a sample of patients with PD and controls, αSyn was detected in all subjects but rates in the PD group were markedly higher [65]. The difference was less marked in those with mild disease, although it remained significant. The sampling and processing techniques used in this study were more sensitive than previous techniques, detecting αSyn even in controls, suggesting a threshold rather than absolute biomarker potential.

### Biomarkers of cell death or dysfunction

#### ^123^I-metaiodobenzylguanidine cardiac scintigraphy

Specialised imaging techniques are already in use to detect cell death and dysfunction in neural systems particularly vulnerable in LB disease. Cardiac ^123^I-metaiodobenzylguanidine (MIBG) scintigraphy uses a noradrenaline analogue to identify presynaptic sympathetic nerve terminals in the heart. This technique can detect cardiac sympathetic denervation that is associated with LB disorders.

In one study, seven asymptomatic individuals had abdominal or pelvic autonomic plexuses removed during surgery (generally for oncologic disease) and examined for the presence of αSyn. Later, the patients underwent ^123^I-MIBG cardiac scintigraphy and a striatal dopamine transporter scan. All four αSyn-positive patients had an abnormal cardiac MIBG scan, compared with none of the αSyn-negative patients. Conversely, only one of four αSyn-positive patients and no αSyn-negative patients had a positive ^123^I-*N*-ω-fluoropropyl-2β-carbomethoxy-3β-(4-iodophenyl)nortropane SPECT scan [37]. Two cases of DLB have been reported with abnormal ^123^I-MIBG cardiac scintigraphy during the MCI phase of the illness [66]. However, cardiac MIBG uptake can be abnormal in congestive cardiac failure, ischaemic heart disease and diabetic autonomic neuropathy, potentially limiting its usefulness, particularly in older people [67].

#### Biomarkers of substantia nigra pathology

**Figure 6:**
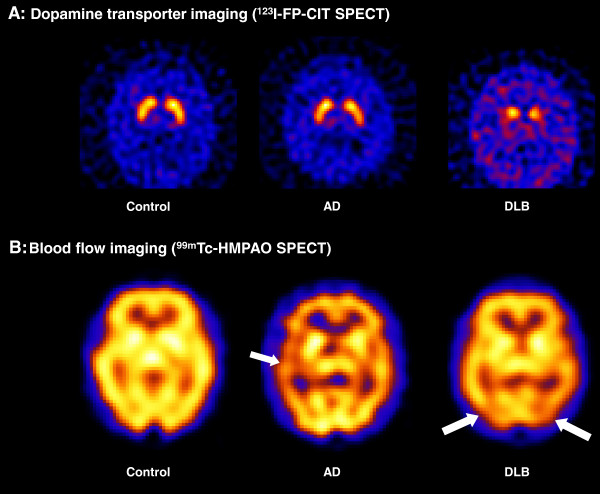
**Examples of imaging abnormalities in dementia with Lewy bodies. (A)** Dopamine transporter imaging. Both the control and Alzheimer’s disease (AD) subjects display normal dopamine transporter levels in the striatum. The dementia with Lewy bodies (DLB) subject displays reduced uptake in the putamen bilaterally, reflecting nigrostriatal degeneration. **(B)** Cerebral blood flow imaging. The AD subject shows decreased perfusion compared with control, particularly in the left temporal lobe. The DLB subject also exhibits perfusion deficits, but these are largely confined to posterior regions including the occipital lobes. FP-CIT, *N*-ω-fluoropropyl-2β-carbomethoxy-3β-(4-iodophenyl)nortropane; HMPAO, exametazime; SPECT, single photon emission computed tomography.

Cell death in the substantia nigra can be assessed by measuring nigrostriatal dopaminergic innervation of the striatum using PET or SPECT with a radiolabelled dopamine analogue (*N*-ω-fluoropropyl-2β-carbomethoxy-3β-(4-iodophenyl)nortropane) (Figure 6). The substantia nigra is a relatively early site of LB deposition (stage 3 in the Braak system) [32] and dopaminergic depletion in the striatum might be anticipated to be a sensitive indicator of early LB disease. However, three of 27 patients with MCI followed-up for 3 years developed DLB, and only one of these had a positive striatal dopamine transporter scan at baseline [68]. Another patient with a positive scan developed frontotemporal dementia. This observation is consistent with the late or minimal involvement of brainstem dopaminergic projection neurones in many DLB cases and suggests that the optimal time point for dopamine transporter imaging may be in established rather than prodromal disease.

Another marker of substantia nigra pathology is enlarged hyperechogenicity on ultrasound. Of 1,535 healthy adults that had baseline substantia nigra ultrasonography and completed follow up, 11 developed PD. Eight of these cases had substantia nigra hyperechogenicity, compared with 18% of the rest of the sample [69]. In an RBD group, 2/43 patients developed DLB. Both had substantia nigra hyperechogenicity at baseline, and one also had reduced striatal dopamine transporters on SPECT imaging [70].

#### Other biomarkers of cell dysfunction or death

Two longitudinal studies following MCI subjects found that DLB subjects had heterogeneous patterns of cortical hypometabolism on ^18^ F-fluorodeoxyglucose PET during the MCI phase, but not the occipital hypometabolism that best differentiates established DLB from AD [71,72]. Fujishiro and colleagues followed a case series of 10 nondemented patients that had attended their memory clinic and were found to have baseline occipital hypometabolism [73]. Four converted to probable DLB, and one to possible DLB; all of these had baseline RBD.

Cortical atrophy is less marked in DLB compared with AD, and therefore is unlikely to be prominent early in the disease [58]. In three autopsy-confirmed DLB patients that had serial magnetic resonance imaging during the MCI phase, hippocampal volumes and rates of hippocampal atrophy were within the range of cognitively normal subjects [21].

### Clinical biomarkers of functional decline

In AD, loss of function – detected by neuropsychological tests or reported by the patient – is expected to appear at the end of the process of protein deposition, cell damage and structural change. Due to the specific topographical development of LB pathology, loss of function in structures affected early in the disease can actually precede the earliest pathological changes in other brain areas (Figure 5B). Damage to the olfactory bulb, the enteric nervous system and midbrain nuclei can lead to anosmia, constipation and RBD, respectively. All of these symptoms have been reported to occur several years before the onset of memory impairment in PD [74] and DLB [29,30]. However, since DLB has an older age of onset than PD, ‘normal’ subjects will be more likely to have symptoms such as constipation, minor motor abnormalities and postural hypotension. This may decrease the specificity of individual clinical biomarkers in prodromal DLB, which may best be described by a pattern of symptoms accumulating over time.

There has been little investigation of clinical biomarkers in MCI cohorts followed up for DLB. One group has intensively studied a cohort with RBD, a population at risk of subsequent development of full-blown LB disease. They found that those with RBD who developed PD or DLB had evidence at baseline of greater postural fall in blood pressure [75], minor motor abnormalities [76] and worse colour vision and olfactory function [77], although not electrocardiographic measures of cardiac autonomic dysfunction [78]. In PD, large cohorts have failed to link later development of PD with baseline orthostatic hypotension or heart-rate variability [36]. The ^13^C-breath test is a measure of delayed gastric emptying that is abnormal even in early PD [79] and could be a marker of prodromal LB disease, although this has not yet been investigated.

**Figure 7:**
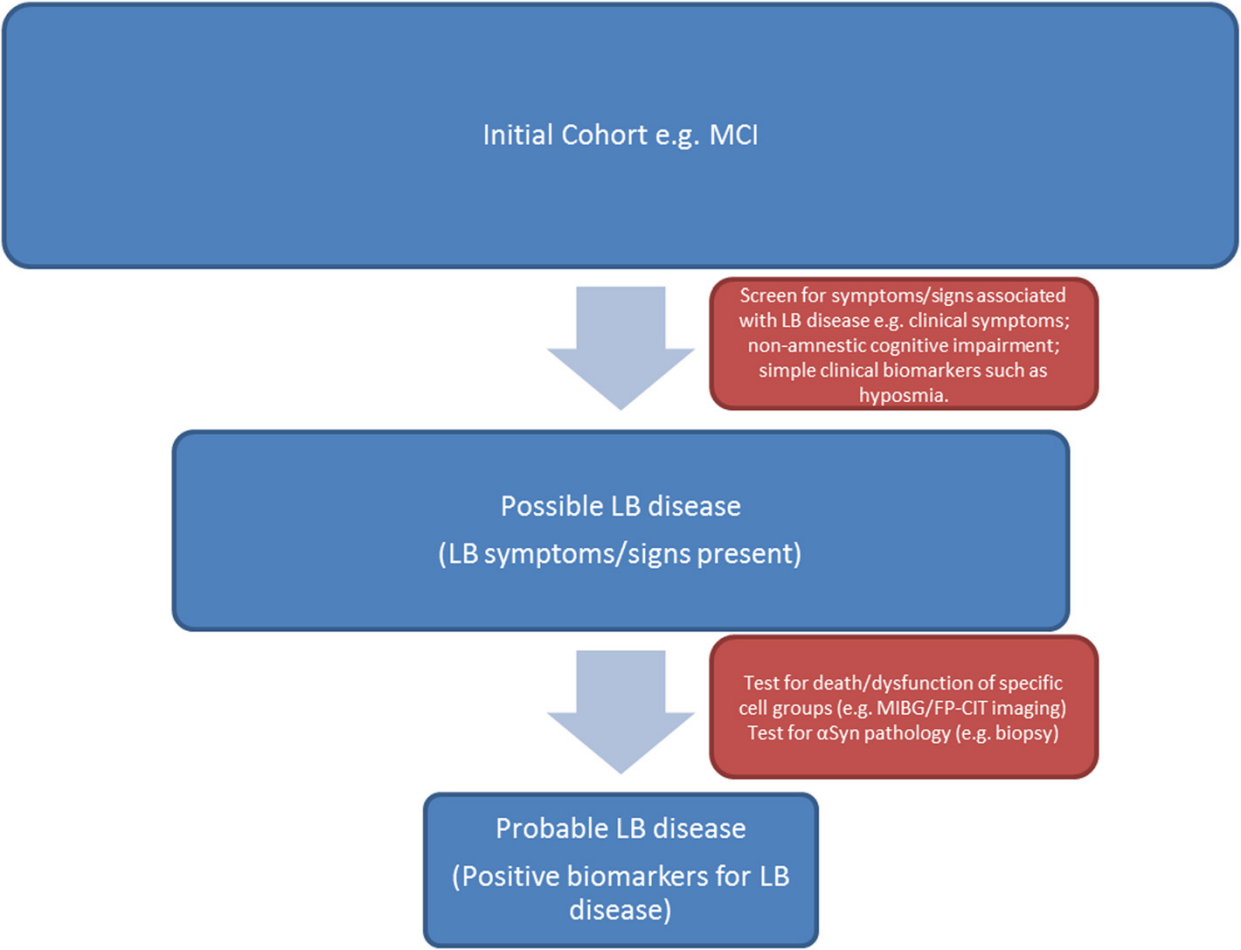
**Hypothetical algorithm for the identification of prodromal Lewy body disease.** The first stage consists of assessment for simple markers of functional decline – symptom questionnaires, cognitive tests and simple clinical biomarkers (for example, biomarkers for hyposmia). In the second stage, those with a profile suggestive of Lewy body (LB) disease would undergo tests with greater specificity, identifying death or dysfunction of cell groups affected by LB pathology, or the presence of alpha-synuclein (αSyn) pathology. This may include cardiac metaiodobenzylguanidine (MIBG) scintigraphy, measures of substantia nigra pathology, such as ultrasound or striatal dopamine transporter imaging, or biopsy for the presence of αSyn. FP-CIT, *N*-ω-fluoropropyl-2β-carbomethoxy-3β-(4-iodophenyl)nortropane; MCI, mild cognitive impairment.

Discovering the true clinical accuracy of symptoms and biomarkers in identifying prodromal DLB is only achievable through further prospective research to determine whether a cumulative risk score or profile could distinguish prodromal LB disease cases from normal controls and other dementia prodromes.A staged approach may be necessary, beginning with bedside screening to identify those that may have LB disease by assessing for markers of functional decline – using symptom questionnaires, cognitive tests and simple clinical biomarkers (for example, biomarkers for hyposmia). In the second stage, those with a profile suggestive of LB disease would undergo tests with greater specificity, identifying death or dysfunction of cell groups affected by LB pathology, or the presence of αSyn pathology. These might include cardiac MIBG scintigraphy, striatal dopamine transporter imaging or a biopsy for the presence of αSyn (Figure 7). The aim of such a process would be to identify cases of prodromal DLB with high sensitivity and specificity, whilst minimising the use of invasive and expensive tests.

Evidence supporting the presence of LB disease may need to be weighed against evidence for the presence of other diseases such as AD. However, amyloid deposition is often seen in DLB [51], and DLB can develop from amnestic MCI [17]. The presence of amnestic MCI and a positive amyloid PET scan, sufficient for a diagnosis of ‘prodromal AD’ or of ‘MCI due to AD – intermediate likelihood’ [80,81], is therefore also consistent with prodromal DLB.

## Issues in prodromal dementia with Lewy bodies research

### Methodological issues

Research into prodromal DLB is still in its early phase. There are some difficulties in studying an entity that has not yet been clearly defined. Retrospective studies of patients with DLB are helpful for assessing symptom development, but these may be susceptible to recall bias and are of limited use in assessing the real clinical utility of biomarkers of prodromal DLB. Longitudinal studies are needed for this, but existing longitudinal studies of unselected MCI groups generally have relatively small numbers of patients that convert to DLB, making such research prohibitively expensive and limiting the conclusions that can be drawn and generalised.

To ameliorate this problem, longitudinal studies need to select at-risk groups. Cohorts of RBD patients are already being investigated in this way [82]. Corresponding cohorts could also be selected based on the presence of other symptoms known to be associated with LB disease, such as hallucinations, parkinsonism or fluctuations, or the presence of nonamnestic cognitive impairments. Examining biomarker and symptom profiles over time in such groups will greatly increase our knowledge of prodromal DLB. In a virtuous cycle, such research will help to refine criteria for identifying at risk groups for subsequent studies.

One must keep in mind that groups with early development of symptoms such as RBD are likely to represent specific subgroups with patterns of disease development that are not necessarily common to all cases of prodromal DLB [83]. It is clear that some cases of DLB develop core features only later in the disease, if at all [84]; these patients may be particularly difficult to identify in the prodromal phase.

### Diagnostic issues

The presence of symptoms associated with LB disease, such as RBD, hyposmia and autonomic dysfunction, along with early biomarkers of LB disease may allow the identification of people who suffer from LB disease prior to the development of cognitive or motor symptoms. DLB is differentiated from PDD on the basis that cognitive features develop before or within a year of parkinsonism. In the move toward earlier diagnosis, the identification of slight parkinsonian signs and mild cognitive difficulties prior to the development of any full clinical syndrome makes such distinctions more difficult. The distinction between DLB and PDD is unlikely to be useful or practicable at this stage and a general classification of ‘prodromal LB disease’ may be more appropriate. Different patterns of biomarkers may possibly emerge as predictors of which patients will progress to develop particular clinical presentations over time.

At present, in the absence of disease-modifying therapies, identifying LB disease prior to the development of cognitive or motor dysfunction has little obvious clinical benefit. However, enquiring about prodromal symptoms may be most helpful in current clinical practice when the clinician is endeavouring to establish the cause of dementia in a patient who already has mild, moderate or even severe impairment but in whom the subtype diagnosis remains unclear. Enquiry about prodromal symptoms, particularly but not restricted to RBD, can be extremely informative in signposting a DLB diagnosis that can be further substantiated with use of a biomarker appropriate to that stage of disease.

## Conclusions

There is a growing belief that by the time dementia has developed, sufficient brain damage has occurred to prevent any disease-modifying treatment working effectively. The identification of prodromal dementia allows researchers to investigate the initial phases of dementia pathophysiology, develop treatments designed to interrupt the development of the dementia syndrome and accurately identify the patients most likely to benefit from these treatments.

Prior to the availability of such treatments, it is not clear that a clinical diagnosis of prodromal dementia is useful to patients or their clinicians [85]. The European Union Joint Action on Alzheimer’s Initiative recently provided recommendations on the timely diagnosis of dementia [86]. Timely diagnosis here reflects ‘access to diagnosis at a time when people can use this information to make sense of what is happening to them, make lifestyle changes and plan for the future’ [86]. Timely diagnosis may thus change when new information or treatments become available. At present, diagnosis of DLB in the early stages of dementia would seem more timely and appropriate than an earlier diagnosis of prodromal LB disease of uncertain prognosis. That said, knowledge of the prodromal development of DLB could help clinicians identify cases of DLB where the diagnosis is uncertain.

For the DLB research community, the diagnosis of prodromal DLB is an area of growing interest, providing an important opportunity to investigate the earliest stages of LB disorders. Such efforts are already underway in RBD [82] and prospective cohorts based upon other risk profiles need to be similarly studied.

Further characterisation of biomarkers confirming the presence of LB pathology has been identified as a priority for LB disease research [87] and is a necessary prerequisite for conducting clinical trials of emerging prevention agents. The widespread deposition of αSyn pathology in DLB may prove key in prodromal diagnosis. Based on current evidence and the temporal development of LB pathology, some of the most promising early biological markers include ^123^I-MIBG cardiac scintigraphy, skin biopsy and measures of substantia nigra pathology. Over the coming years, longitudinal studies should establish which of these markers are most effective. In parallel with this, such studies will help to uncover the early pathophysiology of LB disease, aiding the development of treatment and prevention strategies.

## Note

This article is part of a series on *Lewy Body Dementia*, edited by Ian McKeith and James Galvin. Other articles in this series can be found at http://alzres.com/series/LewyBodyDementia.

## Abbreviations

Aβ: amyloid beta; AD: Alzheimer’s disease; αSyn: alpha-synuclein; DLB: dementia with Lewy bodies; LB: Lewy body; MCI: mild cognitive impairment; MIBG: metaiodobenzylguanidine; PD: Parkinson’s disease; PDD: Parkinson’s disease dementia; PET: positron emission tomography; RBD: rapid eye movement sleep behaviour disorder; SPECT: single photon emission computed tomography.

## Competing interests

The authors declare that they have no competing interests.
